# Mitochondrial composition of and diffusion limiting factors of three social wasp genera *Polistes*, *Ropalidia*, and *parapolybia* (Hymenoptera: Vespidae)

**DOI:** 10.1186/s12862-022-02017-6

**Published:** 2022-05-12

**Authors:** Li Luo, Pan Huang, Bin Chen, Ting-Jing Li

**Affiliations:** Chongqing Key Laboratory of Vector Insects; Chongqing Key Laboratory of Animal Biology; Institute of Entomology and Molecular Biology, Chongqing Normal University, Chongqing, China

**Keywords:** Mitochondrial genome, Geographic distribution, Polistinae, Divergence time

## Abstract

**Background:**

Social wasps *Polistes*, *Ropalidia*, and *Parapolybia*, belonging to the subfamily Polistinae, have obviously different distribution patterns, yet the factors leading to this difference remain unknown.

**Results:**

The 17 newly sequenced mitogenomes of *Polistes*, *Ropalidia*, and *Parapolybia* contain 37 genes, and there are obvious differences among the compositions of the three genera. The monophyly of the genus *Polistes* and a monophyletic Ropalidiini: (*Ropalidia* + *Parapolybia*) are concordant with previous morphological analysis of the subfamily Polistinae. Our inferred divergence time demonstrates *Polistes* (at around 69 Ma) was diverged earlier than *Ropalidia* and *Parapolybia* (at around 61 Ma). The rearrangement of both *trnY* and *trnL1* are shared by all the Polistinae. In addition, the unique rearrangement of TDRL derived at 69 Ma is detected in *Polistes*, and *Ropalidia* contains a Reversal which may derive at 61 Ma. Hereafter, the possibility is elaborated that *Polistes* originated in Aisa and then dispersed from Africa to South America, and *Polistes* and *Ropalidia* spread from Southeast Asia to Australia. At last, continental drift and Quaternary Ice Ages are inferred to be two main limiting factors in the current distributions of the three genera.

**Conclusions:**

Obvious differences occur in the mitochondrial composition of *Polistes*, *Ropalidia*, and *Parapolybia*. According to the reconstructed time-calibrated framework, it is inquired that the continental drifts and the climate are mainly diffusion limiting factors of the three genera.

**Supplementary information:**

The online version contains supplementary material available at 10.1186/s12862-022-02017-6.

## Background

The subfamily Polistinae (Hymenoptera: Vespidae), including more than 950 species of 26 genera and 4 tribes, are social wasps with wide distributions, playing a significant role in the community level and biological control [1–3]. Among the subfamily, *Polistes* Latreille, 1802 is a cosmopolitan and the largest genus with about 300 species [4, 5]. *Ropalidia* Guérin-Méneville, 1831, the third largest genus with more than 200 species, is distributed in a greater part of the Old World with a tropical or subtropical climate [6, 7]. *Parapolybia* de Saussure, 1854, including 13 species, is a small genus and with a much narrower distribution than *Ropalidia* [8]. Meanwhile, only these three genera in the subfamily Polistinae are distributed in China, also with obviously different distribution patterns that *Polistes* is widespread, whereas both *Ropalidia* and *Parapolybia* are mostly distributed to south of the Qinling Mountains–Huai River (QH) line (104° 15′–120° 21′ E, 32° 05′–34° 18′ N). Factors causing their difference of distributions in these three genera are still unknown.

There are many factors that influence the geographical distribution pattern of a species, such as the origin and evolutionary events, the movements of continental plates, the climate of species habitat, the vicissitudes of plant communities, and human activities [3, 9]. Combined with fossil evidence and distribution data of a species, phylogenetic analyses could infer the origin time, evolutionary history, ancestral distribution areas, and the formation history of the present distribution pattern of this species [10]. On the other hand, the early classifications of both *Ropalidia* and *Parapolybia* have undergone relatively extensive transfers based on morphological, behavioral characteristics, as well as partial mitogenomic sequences and nuclear markers (*CO1*, *12 S*, *16 S*, *28 S*, *H3*, and *EF1*-α) [11, 12]. Although sequence data are available, there has been constant debates about which data can yield the most accurate result when the topologies obtained from different data sets conflict [13, 14]. Whereas, the mass applications of whole mitogenome data provide great potential for resolving the phylogeny and biogeography which cannot be solved by one or two mitogenomic sequences or nuclear markers [15]. Mitochondrial genomes (mitogenomes) harbor characteristics of low levels of sequence recombination, short coalescent time, and generally rapid evolutionary rates in both vertebrates and invertebrates; therefore, phylogenetic trees constructed on mitogenomic sequences are always employed to solve the confusions in the origin time, evolutionary history, and distribution pattern [16, 17]. However, the phylogenetic analyses of *Polistes*, *Ropalidia*, and *Parapolybia* have never been studied using whole mitogenome data.

In this study, to explore the origin time of *Polistes*, *Ropalidia*, and *Parapolybia*, as well as to elucidate the factors that contribute to their present distribution patterns, mitogenomes of 17 species belonging to *Polistes*, *Parapolybia*, and *Ropalidia* were sequenced. The gene orders in mitogenomes of the three genera were systematically compared with that in the putative ancestral of Hymenoptera to find the structure and composition which might be related to the distribution patterns [18–20]. And these data, in combination with 18 from previous researches were used to reconstruct phylogenetic trees and estimate origin time (Additional file 1: Table S1). Furthermore, based on the origin time and current distributions, it was analyzed that the vicissitudes of both continental plates and global climates might influence the diversities and distribution patterns of these genera.

## Materials and methods

### Sample collection and DNA extraction

A total of 17 species of these three genera were selected for whole mitogenome sequencing (Additional file 1: Table S1). All the specimens were stored in 95% ethanol prior at − 20 ℃ in Chongqing Normal University (CQNU). Total DNA was extracted from the muscle tissues of thorax using the DNeasy DNA Extraction kit (QIAGEN Shanghai, China). The concentration of double-stranded DNA (dsDNA) in extraction was assayed on a Qubit fluorometer using a dsDNA high-sensitivity kit (Invitrogen Shanghai, China).

### Sequence assembling and analyses

The genomic DNA from each sample was pooled and quantified to be 5.0 µg, and the Illumina TruSeq library was constructed from these DNA, of which the average size of inserted fragment was 480 bp. The library was sequenced on the Illumina Hiseq 2500 platform at Berry Genomics, Beijing; 6 Gb clean data was obtained for each species. These reads were used in de novo assembly with IDBA-UD after getting rid of adapters, unpaired, and lower quality reads by using the NGS QC Toolkit [21, 22]. The parameters used for assemblies with IDBA-UD were a similarity threshold of 98% and minimum and maximum K values of 80 and 240 bp. Using primers designed by Simon [23], the *COX1* and *srRNA* used as targeting sequences were amplified by standard PCR reactions, which were used to confirm if the mitogenomes were assembled from the pooled sequencing files. The BLASTn search was used to identify the mitogenomes sequences based on the reference of bait sequences [24].

PCGs, tRNAs, rRNAs, and control regions were identified by searching homologous sequences against the publicly available Vespidae mitogenomes using ClustalX 1.8 [25]. Nucleotide composition was calculated by using MEGA 6.0 [26]. CG View was used to circularize the mitogenomic sequences [27]. The software package DnaSP 5.0 [28] was used to calculate the non-synonymous (*Ka*) and synonymous (*Ks*) substitution ratio (*Ka*/*Ks*) for each sequenced mitogenome. To test significance of *Ka/Ks* and A + T content among *Polistes*, *Ropalidia*, and *Parapolybia*, the one-way analysis of variance (ANOVA) was used as implemented in R core packages with default settings [29]. Gene rearrangement histories of these three wasp genera were reconstructed by using TreeREx 1.85 [30].

### Phylogenetic analyses

In the phylogenetic analyses, 17 whole mitogenome sequences generated in this study and 18 from previous researches were included, representing the four subfamilies of Vespidae (Additional file 1: Table S1). The mitogenomic sequences of *Apis cerana* (Apidae), *Megachile sculpturalis* (Megachilidae), and *Philanthus triangulum* (Crabronidae) were used as outgroups. Multiple alignments were aligned individually by codon-optimized using the L-INS-i strategy with MAFFT algorithm [31], and ambiguous alignment regions were trimmed from the sequences using Gblocks program in TranslatorX [32, 33]. Alignments of individual genes were concatenated as two datasets: (1) PCGR: 13 PCGs and 2 rRNA; (2) AA: amino acid sequences of the 13 PCGs. The two datasets were used in our phylogenetic analyses. According to the Akaike information criterion (AIC), Partition Finder 2.0 was used to determine the best-fit substitution model (Additional file 1: Table S2) for each gene and the default values for the initial partition settings were applied [34]. The Bayesian inference (BI) with MrBayes v.3.2.7a [35] was performed for phylogenetic inference. The BI tree was constructed with the average deviation of split frequencies below 0.01, approximately 10,000,000 generations were conducted for the matrix, and each set was sampled every 1,000 generations with a burn-in of 25%. Maximum likelihood (ML) analysis was performed by PHYML [36] online web server with default parameters and the node support values were evaluated via a bootstrap test with 100 replicates.

### Divergence time estimation

The divergence time was estimated using BEAST v.2.5.0 [37]. The GTR + I + G nucleotide substitution model and the speciation Yule model were selected as the tree priors with the uncorrelated lognormal relaxed molecular clock model. Two independent Markov Chain Monte Carlo (MCMC) runs, each had a chain length of 1,000,000 generations with sampling every 1000 generations and a first 25% burn-in, were performed to estimate the divergence time. In the fossil calibration database and reported researches, only three subfamilies Eumeninae, Vespinae and Polistinae in the family Vespidae have fossils, among which *Paleovespa menatensis* (64−60 Ma) and *Symmorphus senex* (94−90 Ma) are the most ancient species of Vespinae and Eumeninae fossils, respectively. Accordingly, the two fossils of *P. menatensis* and *S. senex* were selected for calibration in this study [38, 39].

### Distribution and plate distance calculations

The distributions of the three genera based on the latest statistics of species checklists [4, 5, 7, 40–52] were newly sorted out and located on the world map using Bigemap (http://www.bigemap.com). The distance between South America and Africa was calculated according to rates of motion of the South Atlantic Ocean (22–28 mm/a) which based on International Terrestrial Reference Frame 2000 (ITRF2000) [53].

## Results

### The latest distributions of the three genera

**Fig. 1:**
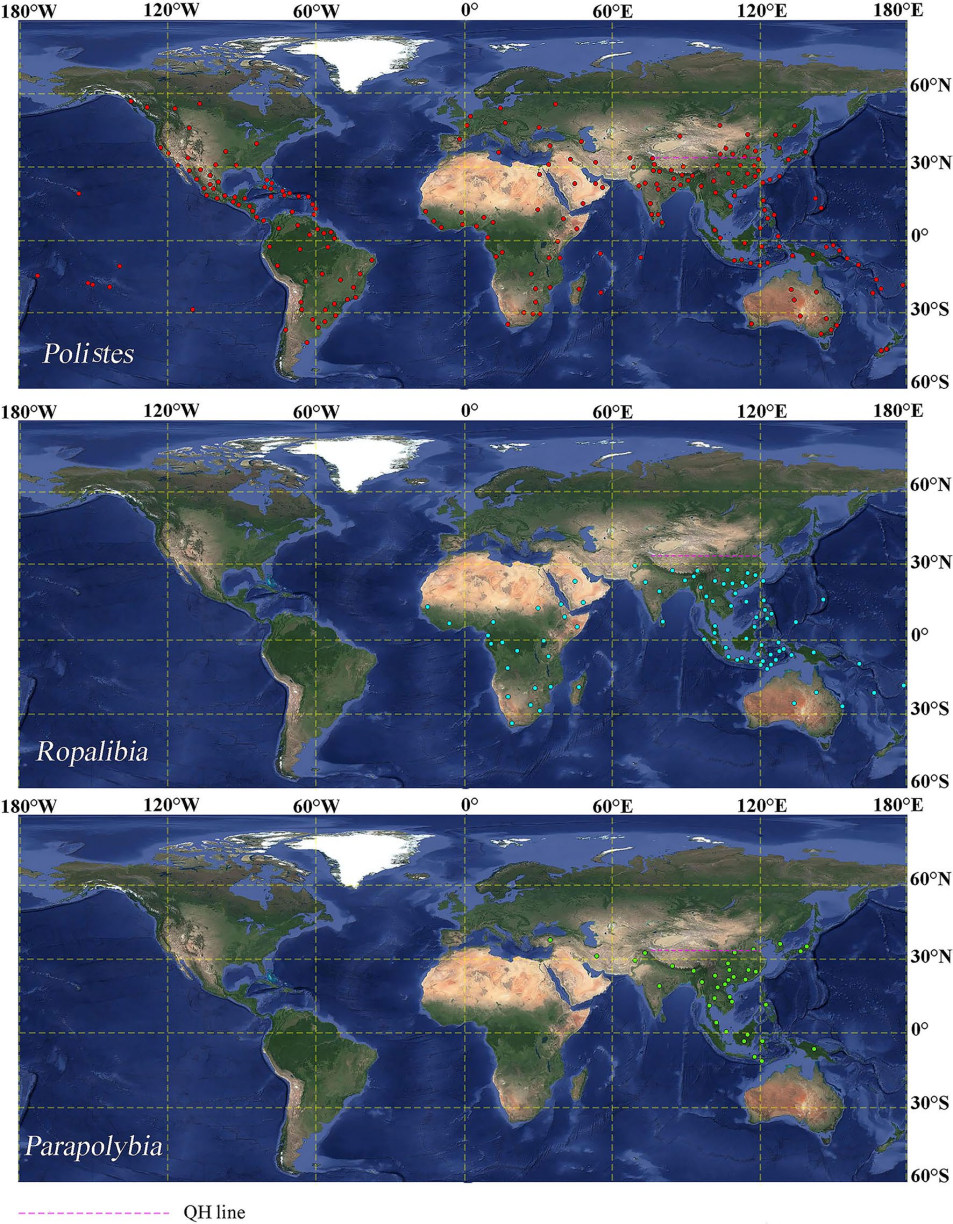
Distributions of *Polistes*, *Ropalidia*, and *Parapolybia*. The purple dashed line represents Qinling Mountains–Huai River (QH) line, and the dots in red, blue, and green represent the recorded distribution locations of *Polistes*, *Ropalidia*, and *Parapolybia*, respectively. The map is made in BigMap, and there are no copyright disputes

The northernmost distributions of *Polistes*, *Ropalidia*, and *Parapolybia* are Victoria of Canada (123°22′ W, 48°25′ N), Pakistan (71° 27′ E, 29° 79′ N), and Turkey (39° 46′ E, 34° 27′ N), respectively. While the southernmost distributions of these three genera are Río Negro of South Africa (63° 03′ W, 40° 82′ S), Western Cape of South Africa (19° 82′ E, 33° 37′ S), and Timor Island (124° 05′ E, 9° 62′ S), respectively (Fig. 1).

### Mitogenomic composition of the three genera

**Fig. 2:**
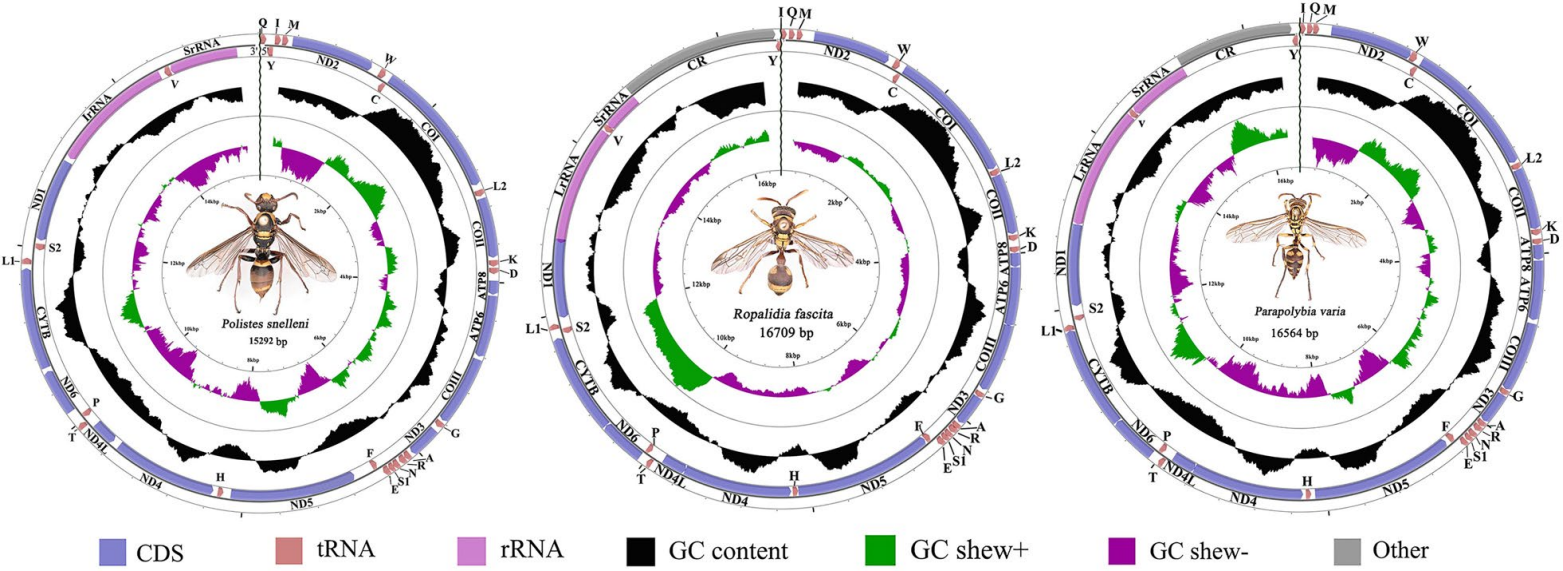
Mitogenomes of *Polistes*, *Ropalidia* and *Parapolybia* sequenced in this study. (In consideration of the almost consistent mitochondrial structure within genus, one inner circle of each genus was only presented in this paper.)

In this study, for the first time, 17 mitogenome sequencing on *Polistes*, *Ropalidia*, and *Parapolybia* was preformed, which greatly enriches the mitogenome data of Vespidae. Most newly sequenced mitogenomes contain 37 genes (Fig. 2), including 13 PCGs, 22 tRNA genes, and two rRNA genes, as well as a control region. However, several mitogenomes are not fully circular molecules due to one or two missing genes, such as *trnY* in *Ropalidoa hongkongensis hongkongensis* and *R*. *variegate*, and *trnQ* in *R. magnanima* are missing. Considering that the other sequenced mitogenomes of the related genera are complete, it is speculated that the above missing genes might be an assembly issue (Additional file 1: Fig. S1).

**Fig. 3:**
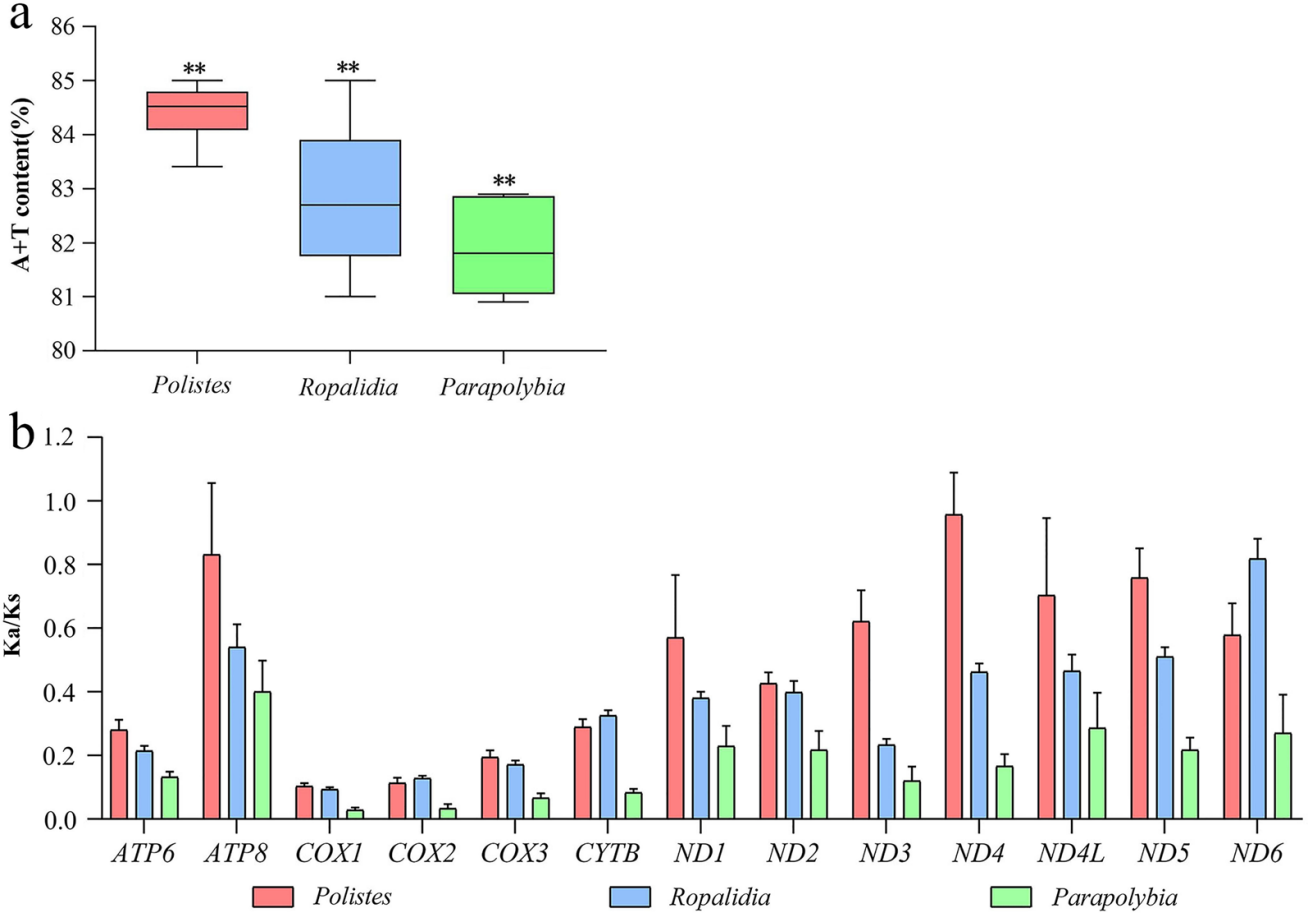
**a** The A + T content (%) of *Polistes*, *Ropalidia* and *Parapolybia* with whole mitogenomes, **P < 0.01; **b** The Ka/Ks values of *Polistes*, *Ropalidia* and *Parapolybia* for 13 PCGs, respectively

Among these three genera, the A + T content of *Polistes* is the highest, and the GC-skew and AT-skew are considerably variable in *Ropalidia* (Additional file 1: Fig. S2). Their nucleotide composition is significantly biased toward adenine and thymine, with an A + T content more than 80% and the order of their A + T content is *Polistes* > *Ropalidia* > *Parapolybia*. And the result of ANOVA suggests that there is a significant difference (P = 0.0020) among the A + T content of the three wasp genera (Fig. 3a).

The results of *Ka/Ks* values of the three genera for 13 PCGs indicate that the order of *Ka/Ks* values is *Polistes* > *Ropalidia* > *Parapolybia* for all PCGs except *ND6*, *COX2* and *CYTB* (Fig. 3b). The *Ka/Ks* values of both *Ropalidia* and *Parapolybia* for 13 PCGs are less than 1 and that of *Polistes* for *ND4* and *ATP8* are more than 1, implying that all PCGs of *Ropalidia* and *Parapolybia* have experienced purifying selection, and to the contrary, *ND4* and *ATP8* of *Polistes* have experienced positive selection. In addition, the *Ka/Ks* values of *COX1* in all three genera are the lowest, indicating that *COX1* is conservative under environmental selection pressure and suitable for molecular bar-coding.

### Phylogenetic relationships and divergence time estimation

In this study, the PCGR and AA datasets were used to individually reconstruct phylogeny of *Polistes*, *Parapolybia*, and *Ropalidia*. Using both BI and ML methods, four phylogenetic trees were generated, and no significant difference was observed in either BI or ML tree between the two datasets. The monophyly of *Polistes*, *Parapolybia* and *Ropalidia* are well supported in all trees with bootstrap values greater than 80 and posterior probabilities of at least 0.89 (Additional file 1: Fig. S3). In addition, within the subfamily Polistinae, the phylogenetic relationship is *Polistes* + (*Ropalidia* + *Parapolybia*). In Vespidae, the phylogenetic analysis shows that the subfamily Stenogastrinae is the sister to all other three subfamilies, and the three remaining subfamilies are divided into two clades: a sister-taxon (Vespinae + Polistinae) and Eumeninae.

**Fig. 4:**
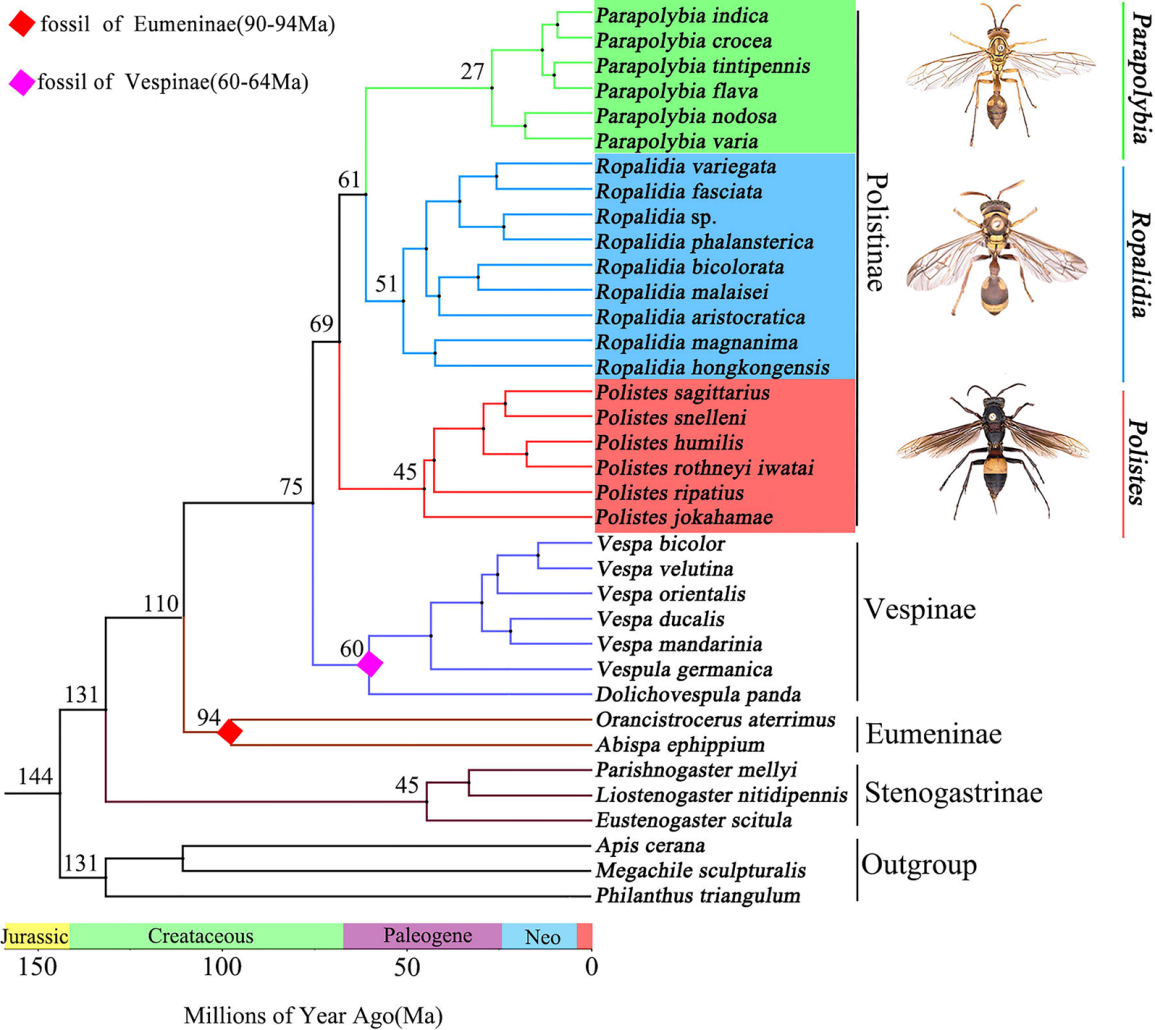
Evolutionary timescale for *Polistes*, *Parapolybia* and *Ropalidia* inferred from PCGR dataset based on two fossil calibration points, the autocorrelated Lognormal relaxed-clock model, the site-heterogeneous mixture GTR + I + G substitution model. A geological time scale is shown at the bottom

Given that the topologies of phylogenetic trees between the two datasets were similar, the PCGR dataset was used to estimate divergence time as it had higher node support values in the initial phylogenetic assessment. The result (Fig. 4) indicates that the subfamily Polistinae evolved over a period of 75 Ma. Meanwhile, the genus *Polistes* diverged at around 69 Ma, *Parapolybia* + *Ropalidia* derived at around 61 Ma, and the origin of *Parapolybia* was later than *Ropalidia.*

### Estimation of the history of rearrangements

**Fig. 5:**
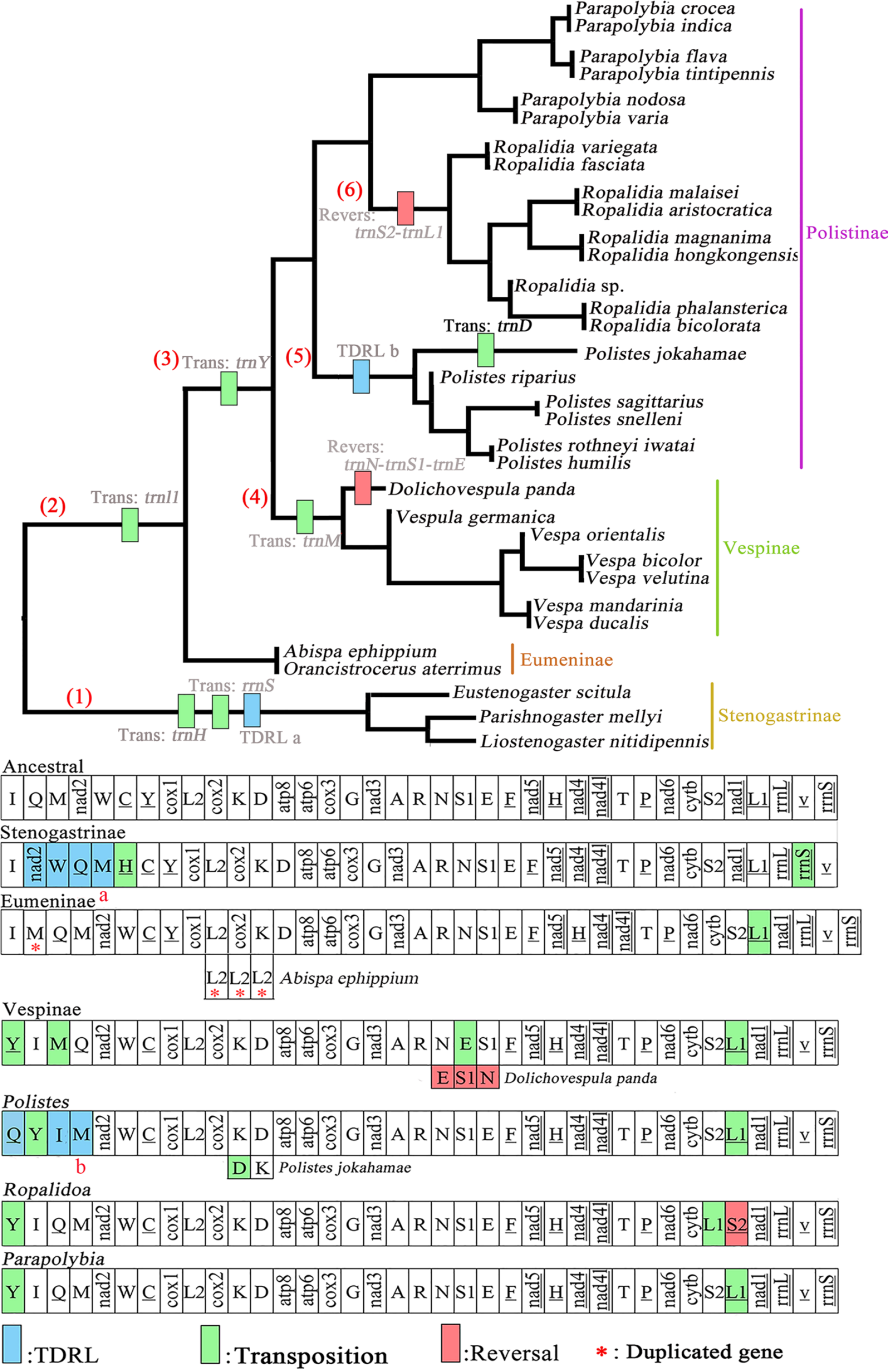
Gene rearrangement history as estimated by TreeREx software. (1)–(6) correspond to evolutionary events discussed in the text. Reves: Revesal; TDRL: Tandem-duplication-random-loss; Trans: Transposition

Reconstructing the pattern of genome rearrangements using the PCGR dataset of the ML phylogenomic topology (Additional file 1: Fig. S3) in TreeREx recovered the following events between the putative ancestral Hymenoptera mitogenome and the four subfamilies of Vespidae (Fig. 5). (1): In the subfamily Stenogastrinae, the transposition of *trnH* is from the location between *trnF* and *nad5* to the upstream of *trnC*, *rrnS* transposes to the upstream of *trnV*, and there is a complicated rearrangement of the genes *trnQ*, *trnM*, *trnW*, and *nad2* which can be explained by tandem-duplication-random-loss (TDRL) model. (2): There is a common event in all other three subfamilies that *trnL1* transposes to the upstream of *nad1*. (3): The transposition of *trnY* from the location between *trnC* and *cox1* to the upstream of *trnI* is observed in both Vespinae and Polistinae. (4): The transpositions of *trnE* and *trnM*, and a reversal of *trnN*-*trnE* occur in Vespinae. (5): The TDRL model of the genes *trnY*, *trnI*, *trnQ*, and *trnM* is detected in all *Polistes*, and the transposition of *trnD* from the downstream of *trnK* to its upstream only occurs in *P. jokahamae*. (6): All species of *Ropalidia* share a reversal *trnS2*-*trnL1*. In addition to the above, duplicated genes *trnM* and *trnL2* are identified in Eumeninae, but gene-duplications are not allowed in TreeREx, so only *trnM* and *trnL2* are coded at a novel position (indicated by asterisk in Fig. 3). Meanwhile, the arrangements of both 13 PCGs and 2 rRNA genes in mitogenomes of Vespidae except the *nad2* and *rrnS* in Stenogastrinae are identical to that of the putative ancestral Hymenoptera.

## Discussion

### Mitogenomic composition

The gene number, the gene composition, codon usage and tRNA secondary structures of the most newly sequenced mitogenomes are similar to other reported mitogenomes of most metazoan animals [54]. However, it was suggested that the variations of GC-skew and AT-skew are related to species biodiversity of geographical distributions with different widths [55]. In this study, *Polistes* is the most widely distributed genus among the three, but it does not show the most significant variation in GC-skew or AT-skew, which may be due to our insufficient sequenced samplings.

### Phylogenetic relationships and divergence time

This present study suggests that all four subfamilies investigated are monophyletic, and the phylogenetic relationships in Vespidae is Stenogastrinae + (Eumeninae + (Vespinae + Polistinae)). A phylogenetic study based on a total of 49 species of Vespidae using transcriptome and target DNA enrichment sequence suggested that the eusocial subfamily Stenogastrinae to be the sister group of all remaining Vespidae, and the subfamily Eumeninae turned out to be paraphyletic [56]. In this study, only two mitogenomes of Eumeninae are contained, which is insufficient to describe the relationship of Eumeninae. In addition, the phylogenetic relationship within the subfamily Polistinae is *Polistes* + (*Ropalidia* + *Parapolybia*), which is supported by previous studies on both morphological characteristics and molecular data [12, 57–60].

It is important to know when *Polistes* originated in Asia because it had been proven that *Polistes* dispersed into the New World from Asia [12]. With most species from Asia, thus, the divergence time of species in this study is mainly limited to this area. Our study indicated that Vespinae and Polistinae separated at 75 Ma and *Polistes* diverged at around 69 Ma, which is consistent with an earlier report [60–62]. This indication is also supported by the most ancient fossil of *Polistes*, namely, *Polistes vergnei* (56–60 Ma). So, it is reasonable that the divergence time of *Polistes* (69 Ma) inferred in this study would not be much older [61].

### Mitogenome rearrangement

Mitogenomes of insects are usually stable in the structure; gene orders are relatively conservative, and recombination events rarely occur in the evolutionary history of insects [63]. However, there are at least eleven rearrangement events of mitogenomes in Vespidae which is consistent with the tendency that the Hymenopteran lineages were clearly toward increasing rearrangement events [64]. Based on the results of the above phylogenetic study, except for the transpositions of both *trnY* and *trnL1* shared by all the Polistinae, *Polistes* contains a more complicated TDRL rearrangement than *Ropalidia* with a reversal rearrangement. It was reported that **s**pecies with the same pattern of mitogenome rearrangement mostly belonged to closely related taxa, which interpreted that they could originate from a common ancestor and then were retained during subsequent lineage diversification [65]. Thus, according to the divergence time estimated in our study, the TDRL of *Polistes’* mitogenomes might arise at 69 Ma, and the reversal of *Ropalidia’* mitogenome might occur at 61 Ma.

### Effects of continental drifts on the three genera

Given the reported fact that the prior existence of land connections cannot explain the present distribution of *Polistes*, the mismatch between the divergence time of *Polistes* and tectonic fragmentation implies an oceanic dispersal [12]. The oceanic dispersal of *Polistes* from Asia to South America may occur in two routes: trans-Atlantic Ocean between South America and Africa, and trans-Pacific Ocean between Asia and South America. Although wasps with wings have the ability of flying, there are few known examples of oceanic dispersal by flight. It is well known that distances are critical for animals to migrate from one island they live on to another one. Up to date, South America is separated from Africa by at least 2,600 km of ocean, and the Atlantic Ocean first appeared well after the onset of the Gondwana breakup (~ 110 Ma) [66]. According to rates of motion of the South Atlantic Ocean (22–28 mm/a) based on International Terrestrial Reference Frame 2000 (ITRF2000) [53], we determined that the distance between South America and Africa at 69 Ma was 902–1148 km, which is consistent with the results of both Sclater [67] and Ford et al. [68]. Correspondingly, South America is separated from Asia by at least 20,000 km of ocean [69]. The Pacific Ocean evolved from Panthalassa in the Late Carboniferous (314–290 Ma), and it had been shrinking owing to the fragmentation of Gondwana, and the expansion of the Indian Ocean and Atlantic Ocean [70], which means South America was separated from Asia by more than 20,000 km of ocean at 69 Ma. On the other hand, oceanic dispersal may also rely on the presence of volcanic islands, floating island, rafting on buoyant vegetation and island hopping. In the Atlantic Ocean, several islands of considerable size (more than 200 km in length) persisted along the present-day submerged Rio Grande Rise and Walvis Ridge at 50 Ma and the long set of islands (at least 800 km in length) had stretched from the Brazilian coast at 20 °S (at the present-day Martin Van Archipelago) at 50–40 Ma [71]. Likewise, there were also many islands in the Pacific Ocean such as Fijian Islands, Borneo, West Sulawesi, and Hawaii Islands, formed by volcanic eruptions in the Late Cretaceous (100–65 Ma) [72]. Among these islands, the closest one to South America called Adamstown was more than 6000 km away, a distance much longer than that between South America and Africa at 69 Ma [73]. Hence, we are inspired to think about the possibility that *Polistes* originated in Asia and then dispersed from Africa to South America via floating islands, the volcanic islands, rafting on buoyant vegetation, and/ or so on, and finally to North America via the Isthmus of Panama between South and North America (Additional file 1: Fig. S4), which was favored by other studies such as rodents, monkeys, birds and some angiosperms [74–77].

*Ropalidia* and *Parapolybia* are distributed in the Old World, and their divergence time is estimated at 61 Ma in the Palaeocene (65–53 Ma). During this period, Africa collided with Europe, leading to the formation of Alps [69]. In addition, even though Indian and Oceania were separated from the Gondwanaland during the Late Jurassic (~ 135 Ma), Indian also collided with Asia in Southern Tibet in the Eocene (53–36.5 Ma). Hereafter, all the continents of the Old World except Australia had been connected until the present day [78]. According to the formation history of the Old World, there should be chances that *Ropalidia* and *Parapolybia* spread among various plates except Australia. Because there were many islands, such as Philippine Islands and New Guinea, between Australia and the Eurasian subcontinent at 65–53 Ma [69], it is possible that the two genera *Ropalidia* and *Parapolybia* spread to Australia through these islands. Our inference is also supported by the result of Saito et al. that *Ropalidia* in Australia actually originated in New Guinean and migrated via the Cape York Peninsula and further speciated in Australia [79]. In this study, the questions that where *Ropalidia* and *Parapolybia* originated and why *Parapolybia* species are not recorded in Australia remain unanswered, and require further information.

### Effects of climate changes on the three genera

Climate changes also have major impacts on terrestrial biodiversity, directly affecting species distribution patterns [80, 81]. In our study, the last major climate change is traced back to the start of Quaternary Ice Ages (2.6–0.015 Ma) [82], because the current entire northern fauna and flora had been greatly changed during Quaternary Ice Ages when the European ice sheets covered the lands in Europe including Siberia to Mongolia, and the North American ice sheets moved from Canada to the northern United States, where it was extremely cold that many animals and plants had to move to the south [82, 83]. And the current climate had not been formed until the end of the Pleistocene (~ 0.015 Ma) [82, 84]. The last Ice Age ended at the beginning of the Holocene (0.015 Ma), and then the climate in the Holocene (0.015 Ma–present) became warmer, which was similar to the present-day climate [85]. As the climate getting warmer, some animals and plants migrated backward to the north [86]. Currently, there are a few *Polistes* and few of both *Ropalidia* and *Parapolybia* distributed in the areas covered by ice sheets during Quaternary Ice Ages. Therefore, it may be inferred that Quaternary Ice Ages was a limiting factor in the northernmost distributions of the three genera.

Furthermore, it has been indicated that temperatures are important in defining the scope for intrasexual signaling in social wasps and play a role in maintaining variations in intrasexual traits in the view of sexual selection [87]. The most suitable temperature for social wasps is 25 ℃ and the most comfortable relative humidity is 50–70% [88, 89]. The average annual climate in Antarctica is − 25 ℃ and the highest temperature in the North Pole area is − 8 ℃ [90], which may be one of the reasons that the wasps are not distributed there. There is a long winter and short summer in the middle temperate zone (40° N to 50° N, 40° S to 50° S) [91], including Mongolia Plateau, most of Northeast China, and North Canada, where annual temperatures vary greatly and the living animals are mostly homoiothermic animals [92]. In view of the influence of the temperature, most of wasps have the habit of overwintering [91], and long-term exposure to low temperatures would result in failure of wasps to overwinter [92], so there are relatively fewer *Polistes* and neither of *Ropalidia* and *Parapolybia* distributed in the middle temperate zone. To the contrary, in the subtropics (23°26’ N to 40° N, 23°26’ S to 40° S) and tropics (23° 26′ S to 23° 26′ N ) [93], there is a small difference in the annual temperatures (above zero in winter), and plentiful precipitation and vegetation can support sufficient foods [94, 95]. Therefore, modern climate pattern also plays an important role in these genera distributions, as most of these wasps are coincidentally distributed in tropical and subtropical zones.

Meanwhile, the fact that *Polistes*, *Ropalidia*, and *Parapolybia* are abundant to the south of QH line in China can also be explained by our finding that the climate pattern influences species distributions. QH line is situated across the middle temperate, tropical and subtropical zones and is the north-south geographical, climatic, and demographic watershed of China. And it also serves as the boundary of the middle temperate and subtropical zones, and the boundary of the Palearctic and Oriental regions [96].

## Conclusions

Our results reveal relatively comprehensive mitochondrial genome features of the three genera *Polistes*, *Ropalidia*, and *Parapolybia*, but due to the limited number and distributions of samples, some features, such as the relationship between GC-skew/AT-skew and species biodiversity of geographical distributions require further data analysis. At the same time, the analysis of the oceanic dispersal of the genus *Polistes* still requires more data to determine the respective origin time when *Polistes* derived in Africa and South America, and to clarify the specific diffusion path. And other potentially limiting factors in the current distributions of the three genera need to be further explored, such as human activities, plant communities, and so on.

## Supplementary Information

**Additional file 1.** **Table S1. **The information of mitochondrial genomes in thisstudy. **Table S2.** The best partitioning schemeselected by PartitionFinder for different data matrices. **Fig.S1.** Mitogenome organization of *Polistes*, *Parapolybia*and* Ropalidia*referenced with the ancestral insect mtgenomes. Theunderlined symbols are located on the N-strand and others on the J-strand. Theyellow, blue and green blocks denote tRNAs, PCGs and control regions,respectively. The red font means rearranged genes. **Fig. S2.** TheA+T content (%), AT-skew, G+C content (%) and GC-skew of the *Polistes*, *Ropalidia*and *Parapolybia* whole mitogenomes. **Fig. S3.** Reconstruction of phylogenetic tree determined by Bayesianinference and Maximum Likelihood methods based on PCGR and AA datasets ofVespidae mitogenomes. Bayesian posterior probabilities (left) and Parsimonybootstrap (right) are shown at relevant branches of the ML tree. **Fig. S4. **The proliferation route of *Polistes*from the Old World to the New World. Southeast Asia as the ancestor region of *Polistes* in New World is marked by a star. Transatlanticroutes of invasion are shown in solid red line, potential transpacific routes of invasion are shown inblue dashes. The green dot only representsits continent rather than any specific location. The map is made in BigMap, and there are no copyright disputes.

## Data Availability

Mitochondrial genome sequences are accessible on GenBank and accession numbers were in electronic supplementary material of Additional file 1: Table S1.

## Declarations

This study does not violate any copyright or other personal proprietary right of any person or entity and it contains no abusive, defamatory, obscene or fraudulent statements, nor any other statements that are unlawful in any way.
