# Supplementary material for: Mitochondrial composition of and diffusion limiting factors of three social wasp genera Polistes, Ropalidia, and parapolybia (Hymenoptera: Vespidae)
Source: BMC Ecol Evol. 2022 May 12;22:63. doi: 10.1186/s12862-022-02017-6 (PMC9097357; doi:10.1186/s12862-022-02017-6)
Supplement: Supplementary file 1 — Additional file 1. Table S1. The information of mitochondrial genomes in thisstudy. Table S2. The best partitioning schemeselected by PartitionFinder for different data matrices. Fig.S1. Mitogenome organization of Polistes, Parapolybiaand Ropalidiareferenced with the ancestral insect mtgenomes. Theunderlined symbols are located on the N-strand and others on the J-strand. Theyellow, blue and green blocks denote tRNAs, PCGs and control regions,respectively. The red font means rearranged genes. Fig. S2. TheA+T content (%), AT-skew, G+C content (%) and GC-skew of the Polistes, Ropalidiaand Parapolybia whole mitogenomes. Fig. S3. Reconstruction of phylogenetic tree determined by Bayesianinference and Maximum Likelihood methods based on PCGR and AA datasets ofVespidae mitogenomes. Bayesian posterior probabilities (left) and Parsimonybootstrap (right) are shown at relevant branches of the ML tree. Fig. S4. The proliferation route of Polistesfrom the Old World to the New World. Southeast Asia as the ancestor region of Polistes in New World is marked by a star. Transatlanticroutes of invasion are shown in solid red line, potential transpacific routes of invasion are shown inblue dashes. The green dot only representsits continent rather than any specific location. The map is made in BigMap, and there are no copyright disputes. [file 12862_2022_2017_MOESM1_ESM.doc]

**Additional file**

**Table. S1** The information of mitochondrial genomes in this study.

|  | Species | Genus | | | Collection locations | | Accession number |
| --- | --- | --- | --- | --- | --- | --- | --- |
| Ingroup | *Polistes sagittarius* | | *Polistes* | China | | MT948192 | |
|  | *Polistes rothneyi iwatai* | | *Polistes* | China | | MT948194 | |
|  | *Polistes snelleni* | | *Polistes* | China | | MT948193 | |
|  | *Polists humilis* | | *Polistes* | Australia | | EU024653 | |
|  | *Polistes jokahamae* | | *Polistes* | China | | KR052468 | |
|  | *Polistes riparius* | | *Polistes* | Japan | | LC519884 | |
|  | *Ropalidia* sp | | *Ropalidia* | Madagascar | | MK034142 | |
|  | *Ropalidia phalansterica* | | *Ropalidia* | Madagascar | | MK034143 | |
|  | *Ropalidia bicolorata* | | *Ropalidia* | China | | MK034144 | |
|  | *Ropalidia fasciata* | | *Ropalidia* | China | | MK034145 | |
|  | *Ropalidia magnanima* | | *Ropalidia* | China | | MK034146 | |
|  | *Ropalidia hongkongensis* | | *Ropalidia* | China | | MK034147 | |
|  | *Ropalidia variegata* | | *Ropalidia* | China | | MK034148 | |
|  | *Ropalidia malaisei* | | *Ropalidia* | China | | MK034149 | |
|  | *Ropalidia aristocratica* | | *Ropalidia* | China | | MK034150 | |
|  | *Parapolybia varia* | | *Parapolybia* | China | | MH065751 | |
|  | *Parapolybia tintipennis* | | *Parapolybia* | China | | MH065754 | |
|  | *Parapolybia flava* | | *Parapolybia* | China | | MH065755 | |
|  | *Parapolybia nodosa* | | *Parapolybia* | China | | MH065756 | |
|  | *Parapolybia indica* | | *Parapolybia* | China | | MH065757 | |
|  | *Parapolybia crocea* | | *Parapolybia* | China | | KY679828 | |
|  | *Vespa mandarinia* | | *Vespinae* | China | | NC_027172 | |
|  | *Vespula germanica* | | *Vespinae* | China | | KR703583 | |
|  | *Vespa bicolor* | | *Vespinae* | China | | KJ735511 | |
|  | *Vespa velutina* | | *Vespinae* | Korea | | NC_035146 | |
|  | *Vespa orientalis* | | *Vespinae* | Jordan | | KY563657 | |
|  | *Vespa ducalis* | | *Vespinae* | Jordan | | KX950825 | |
|  | *Dolichovespula panda* | | *Vespinae* | China | | NC_036067 | |
|  | *Abispa ephippium* | | *Eumeninae* | Australia | | NC011520 | |
|  | *Orancistrocerus aterrimus* | | *Eumeninae* | China | | KY941926 | |
|  | *Eustenogaster scitula* | | *Stenogastrinae* | China | | KY856830 | |
|  | *Liostenogaste rnitidipennis* | | *Stenogastrinae* | China | | MH910629 | |
|  | *Parishnogaster mellyi* | | *Stenogastrinae* | China | | MH910630 | |
| Outgroup | *Apis cerana* | | *Apoidea* | China | | NC014295 | |
|  | *Megachile sculpturalis* | | *Apoidea* | China | | NC028017 | |
|  | *Philanthus triangulum* | | *Sphecoidea* | Germany | | NC017007 | |

**Table. S2** The best partitioning scheme selected by PartitionFinder for different data matrices.

| Data matrices | Optimal Partition | Model | Initial Partition | Optimal Partition | Model | Initial Partition |
| --- | --- | --- | --- | --- | --- | --- |
| PCG-codon partition | Partition 1 | GTR+I+G | *a6p1* | Partition 9 | GTR+I+G | *n1p1* |
|  | Partition 2 | GTR+I+G | *c3p2, cbp2, a6p2* | Partition 10 | GTR+G | *n2p1* |
|  | Partition3 | GTR+I+G | *c3p1, a8p1, c2p1* | Partition 11 | GTR+G | *n6p2, n2p2* |
|  | Partition 4 | GTR+I+G | *a8p2, n4p2, n1p2* | Partition 12 | GTR+G | *n3p1, n6p1* |
|  | Partition 5 | GTR+I+G | *c1p1* | Partition 13 | GTR+I+G | *n5p1, n4p1* |
|  | Partition6 | GTR+I+G | *c1p2* | Partition 14 | GTR+G | *n4lp1* |
|  | Partition 7 | GTR+I+G | *n3p2, c2p2* | Partition 15 | GTR+G | *n4lp2* |
|  | Partition 8 | GTR+I+G | *cbp1* | Partition 16 | GTR+I+G | *n5p2* |
| PCGR-codon partition | Partition 1 | GTR+I+G | *a6p1* | Partition 10 | GTR+G | *n2p1* |
|  | Partition 2 | GTR+I+G | *c3p2, cbp2, a6p2* | Partition 11 | GTR+G | *n6p2, n2p2* |
|  | Partition3 | GTR+I+G | *c3p1, a8p1, c2p1* | Partition 12 | GTR+ G | *n3p1, n6p1* |
|  | Partition 4 | GTR+I+G | *a8p2, n4p2, n1p2* | Partition13 | GTR+I+G | *n5p1, n4p1* |
|  | Partition 5 | GTR+I+G | *c1p1* | Partition 14 | GTR+ G | *n4lp1* |
|  | Partition6 | GTR+I+G | *c1p2* | Partition 15 | GTR+G | *n4lp2* |
|  | Partition 7 | GTR+I+G | *n3p2, c2p2* | Partition 16 | GTR+I+G | *n5p2* |
|  | Partition 8 | GTR+I+G | *cbp1* | Partition17 | GTR+G | *rrnl, rrnS* |
|  | Partition 9 | GTR+I+G | *n1p1* |  |  |  |


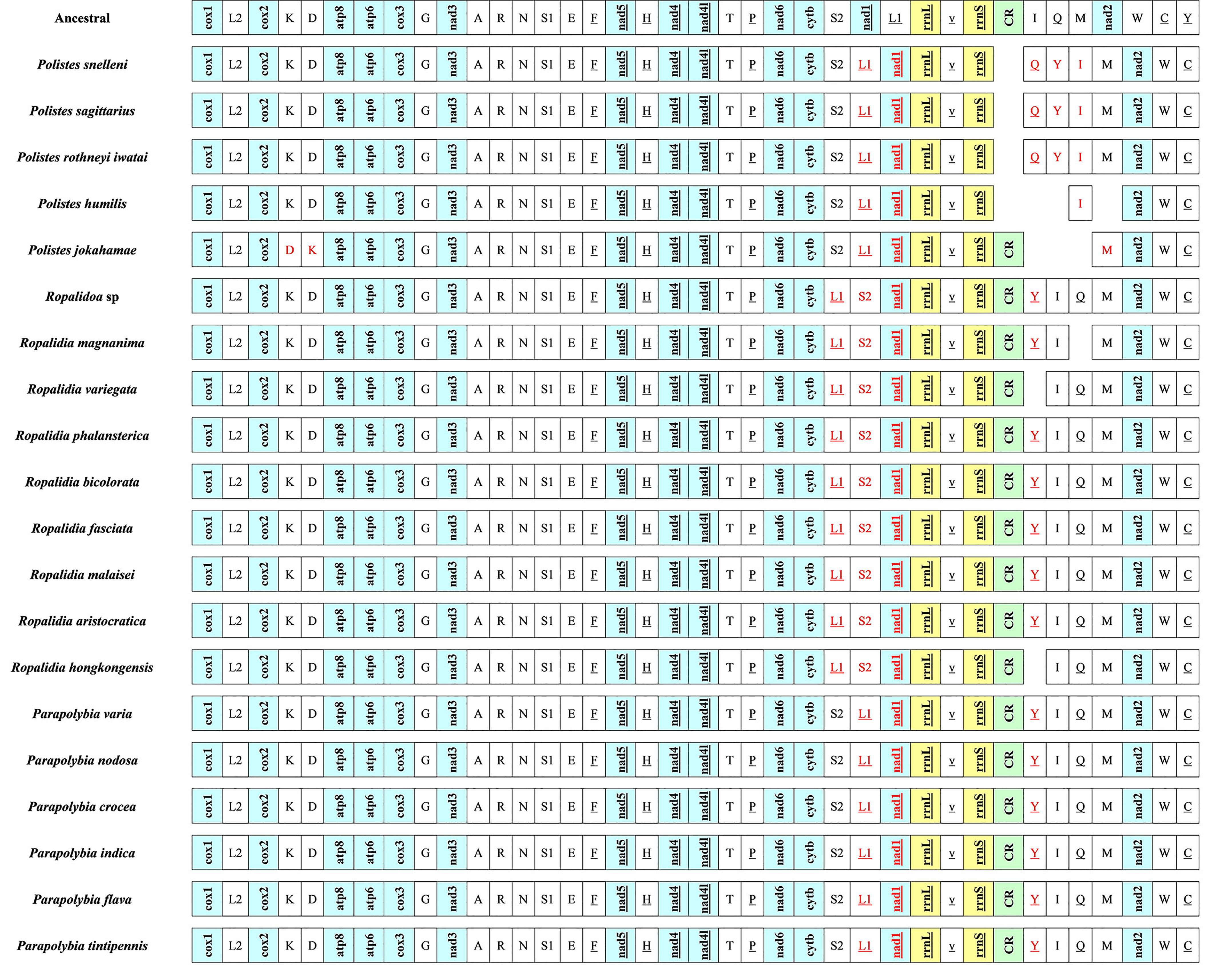


**Fig. S1** Mitogenome organization of *Polistes*, *Parapolybia* and *Ropalidia* referenced with the ancestral insect mtgenomes. The underlined symbols are located on the N-strand and others on the J-strand. The yellow, blue and green blocks denote tRNAs, PCGs and control regions, respectively. The red font means rearranged genes.


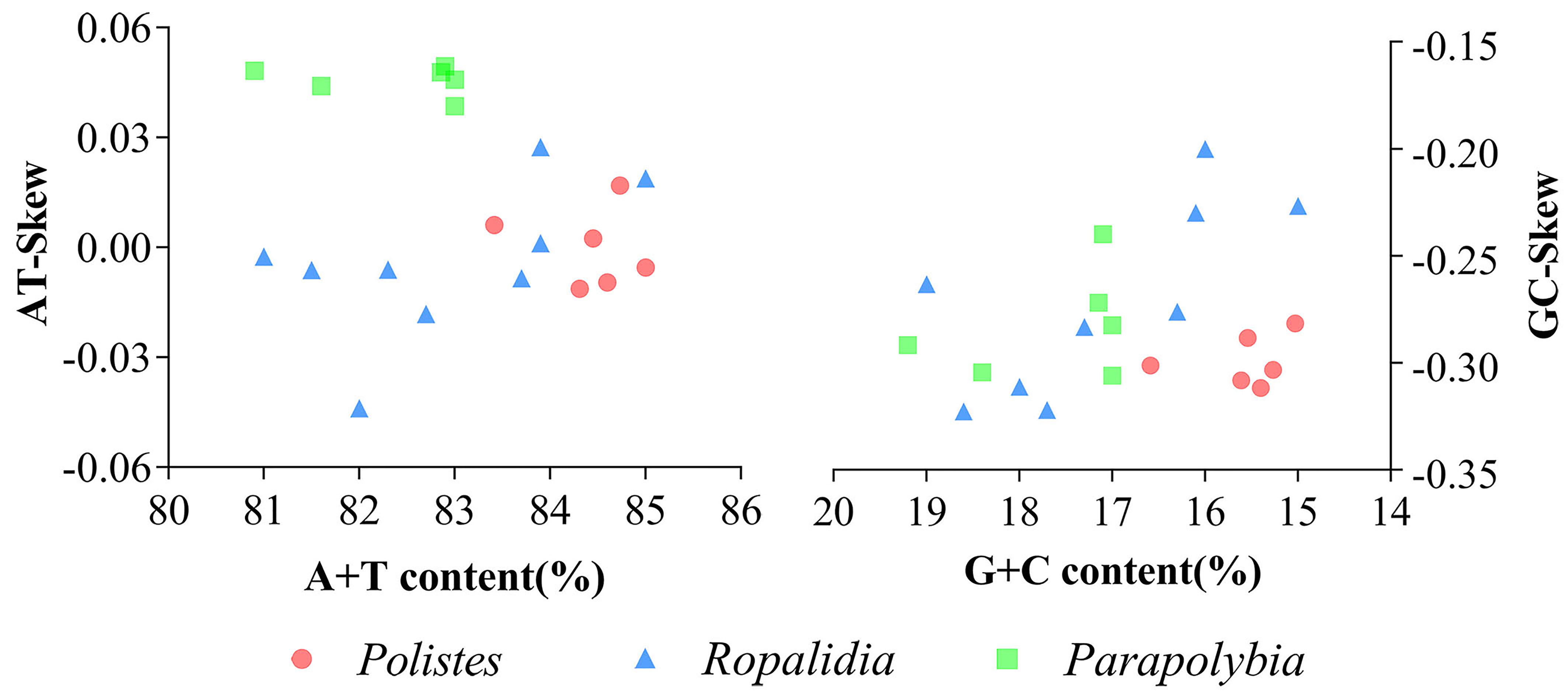


**Fig. S2** The A+T content (%), AT-skew, G+C content (%) and GC-skew of the *Polistes*, *Ropalidia* and *Parapolybia* whole mitogenomes.


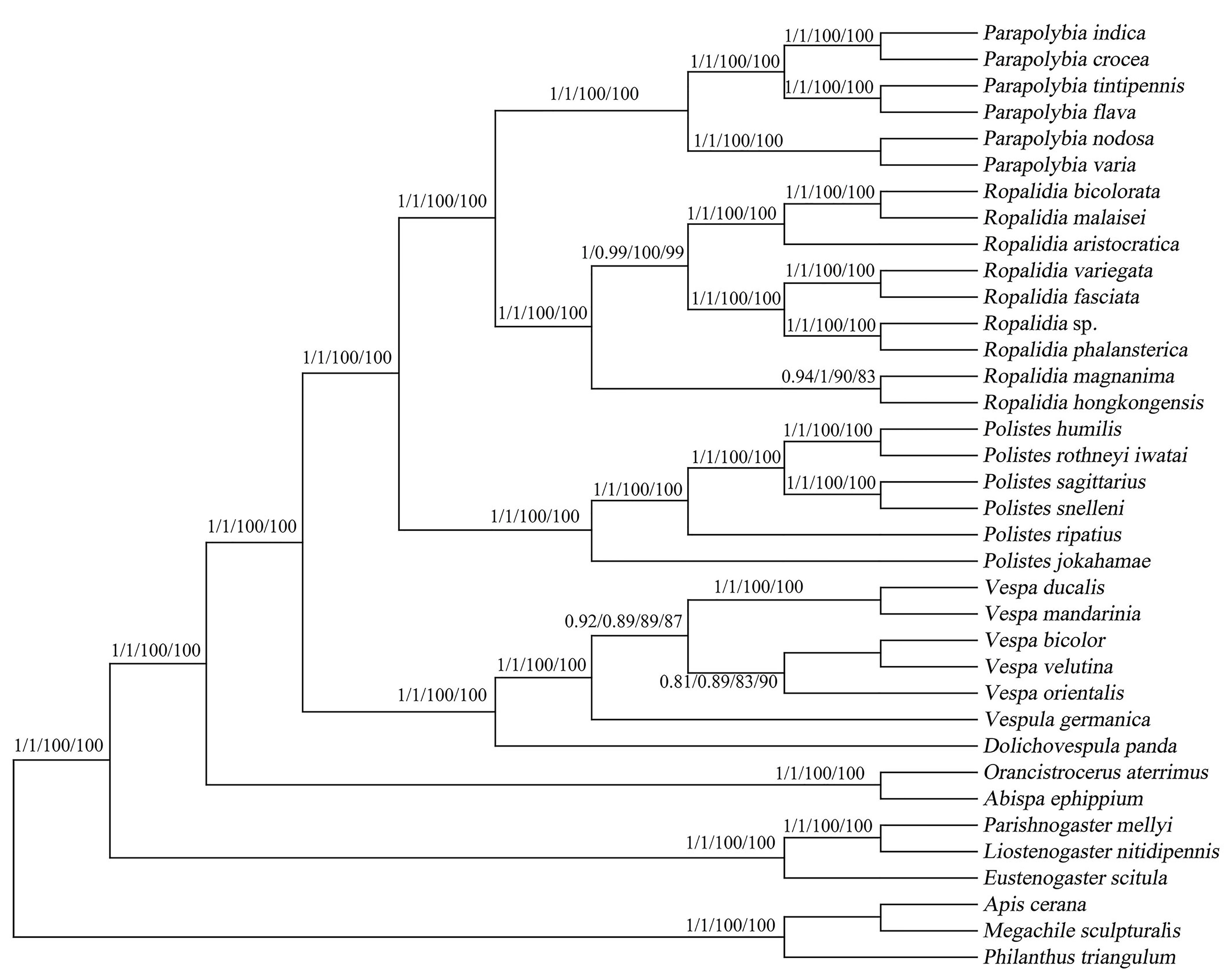


**Fig. S3** Reconstruction of phylogenetic tree determined by Bayesian inference and Maximum Likelihood methods based on PCGR and AA datasets of Vespidae mitogenomes. Bayesian posterior probabilities (left) and Parsimony bootstrap (right) are shown at relevant branches of the ML tree.


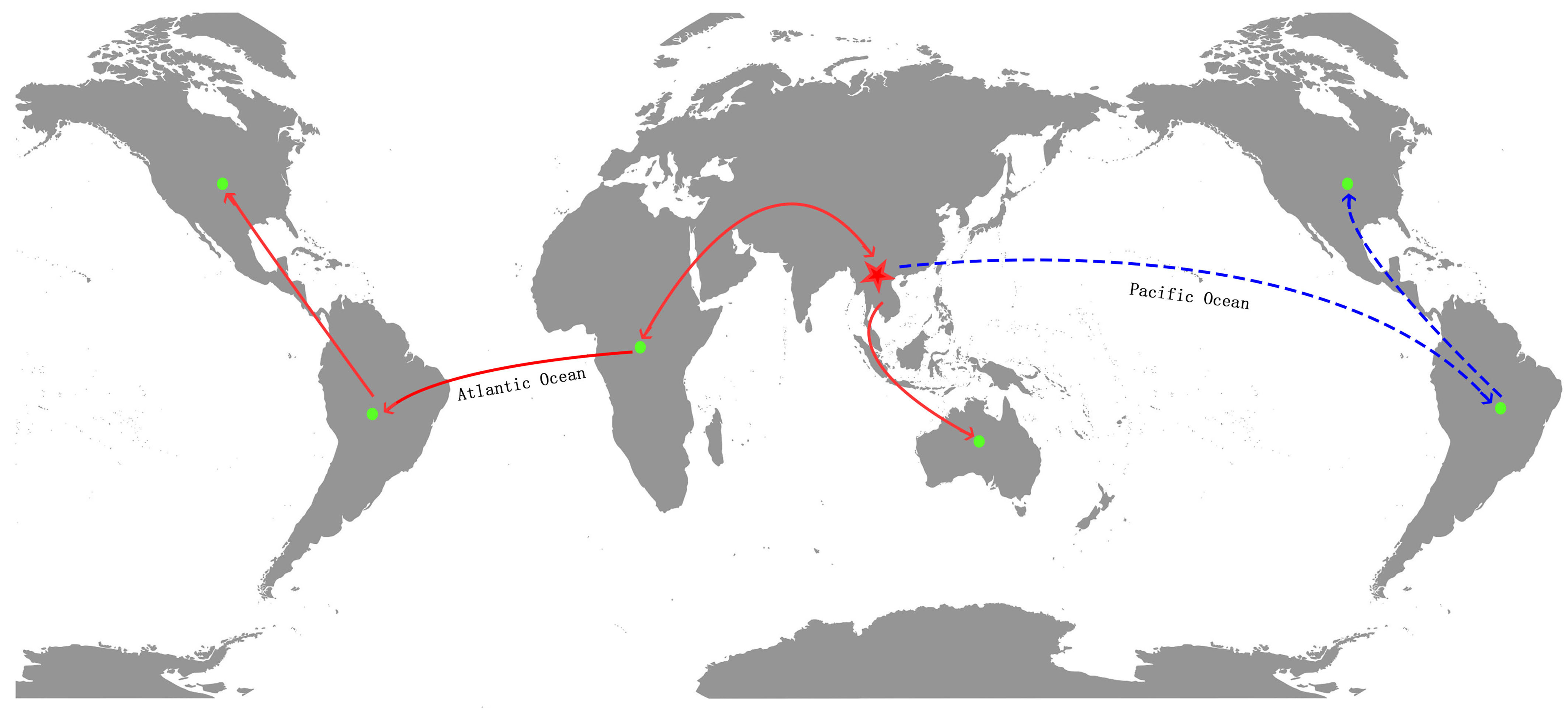


**Fig. S4** The proliferation route of *Polistes* from the Old World to the New World. Southeast Asia as the ancestor region of *Polistes* in New World is marked by a star. Transatlantic routes of invasion are shown in solid red line, potential transpacific routes of invasion are shown in blue dashes. The green dot only represents its continent rather than any specific location. The map is made in BigMap, and there are no copyright disputes.
